# Drug-Induced Gingival Hyperplasia in a Hypertensive Patient: A Case Report

**DOI:** 10.7759/cureus.34558

**Published:** 2023-02-02

**Authors:** Sanket S Bakshi, Mahak Choudhary, Aman Agrawal, Swarupa Chakole

**Affiliations:** 1 Department of Medicine, Jawaharlal Nehru Medical College, Datta Meghe Institute of Medical Sciences, Wardha, IND; 2 Community Medicine, Datta Meghe Institute of Medical Sciences, Wardha, IND

**Keywords:** lymphocytosis, mucosal membrane, gingivitis, hypertension, amlodipine

## Abstract

Hypertension is one of the most notorious non-communicable diseases the medical fraternity is dealing with in this decade. A wide array of medications have been included in the treatment regimen, one of which is calcium channel blockers. Amlodipine is commonly administered from this class. The reports of adverse drug reactions to the intake of amlodipine are very scarce to date. Association of gingival hyperplasia with the administration of this drug is rare and is what we have reported in this case. The theory that is being put forward for this adverse reaction is that the gingival fibroblasts are induced via the proliferative signaling pathways in association with the formation of bacterial plaques. Several classes of drugs other than calcium channel blockers are known to cause this reaction. Anti-epileptics along with anti-psychotic drugs are comparatively more prevalent. Thorough scaling and root planing are used to identify and treat amlodipine-induced gingival hypertrophy. The cause of gingival expansion is unknown, and there is currently no cure other than surgically removing the enlarged tissue and maintaining better dental hygiene. Immediate stoppage of the causative drug is advised in these cases along with the surgical remodeling of the affected gingiva.

## Introduction

Gum hypertrophy and gum hyperplasia are synonyms that refer to the same histological condition of enlarged gums. There are several reasons for gum growth. It has been proven that a patient's genetic predisposition causes drug-induced enlargement. More than 20 pharmaceutical drugs are now linked to gingival hypertrophy [1]. Anticonvulsants, immunosuppressives, and antihypertensive medicines are the three primary pharmacological classes. Seymour et al. initially noted gingival overgrowth as a side effect of amlodipine, a more recent dihydropyridine drug used to treat hypertension and angina. Within two months of commencement, Lafzi et al. found that patients receiving 10 mg of amlodipine daily experienced fast development of gingival hyperplasia [2,3]. Within one to three months of starting therapy with the linked medicine, gingival expansion commonly manifests clinically. In the United States and around the world, hypertension is one of the main causes of mortality and disability [1,4]. While nurse practitioners assist patients in managing their blood pressure with medication, physicians provide lifestyle counselling. Calcium channel blockers, including amlodipine, are among the first-line treatments for hypertension. Amlodipine has about 70 million prescriptions written for it annually in the US [5]. Gingival overgrowth, if left unchecked, can result in tooth movement and ultimately loss. Additionally, dental modifications lead to discomfort, financial difficulty, and psychological trauma. Due to restricted access to dental treatment, underserved patients are substantially more likely to have these harmful effects [6]. Affordable therapy consisting of first-line drugs has been proven to be beneficial, considering the rising incidence of hypertension, especially in the rural population [7]. 

## Case presentation

**Figure 1:**
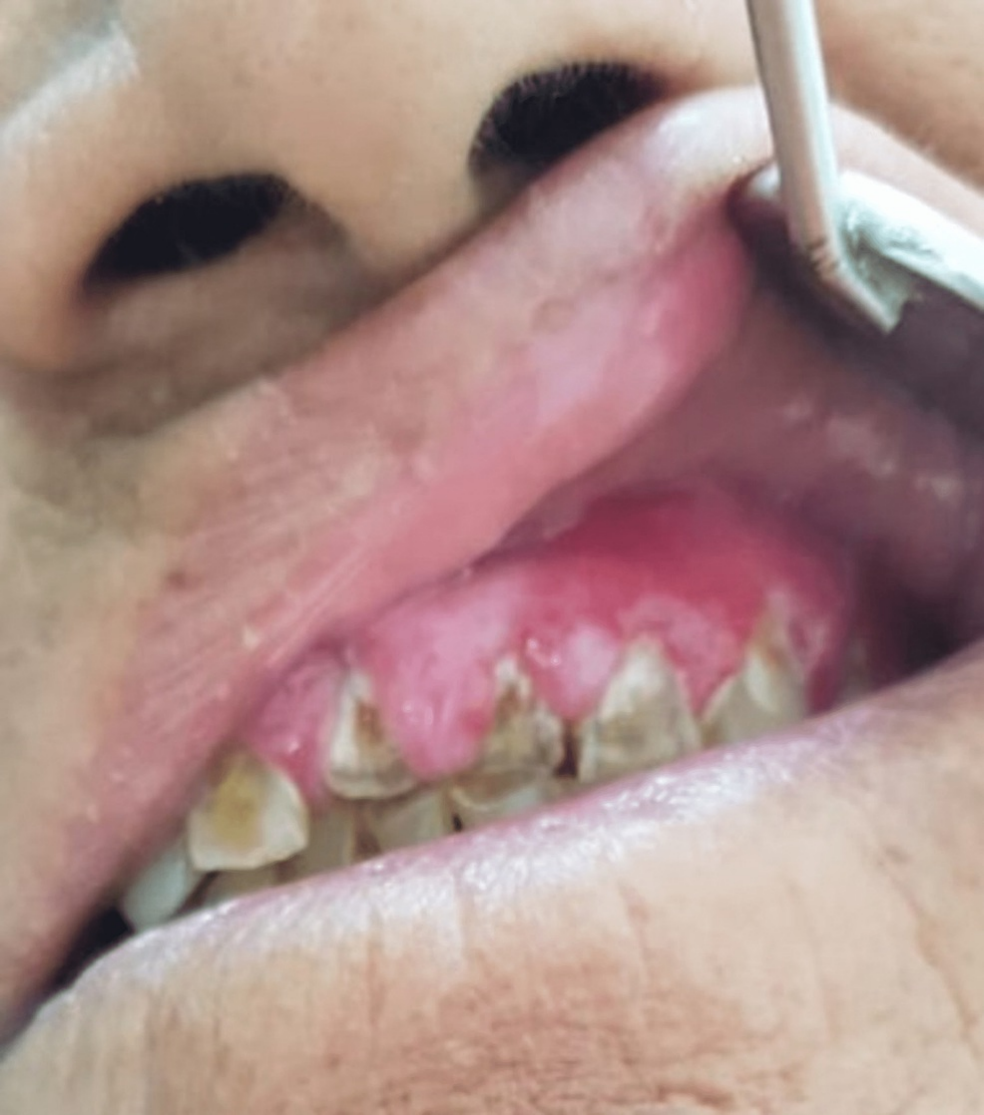
Gingival hypertrophy after amlodipine administration

**Figure 2:**
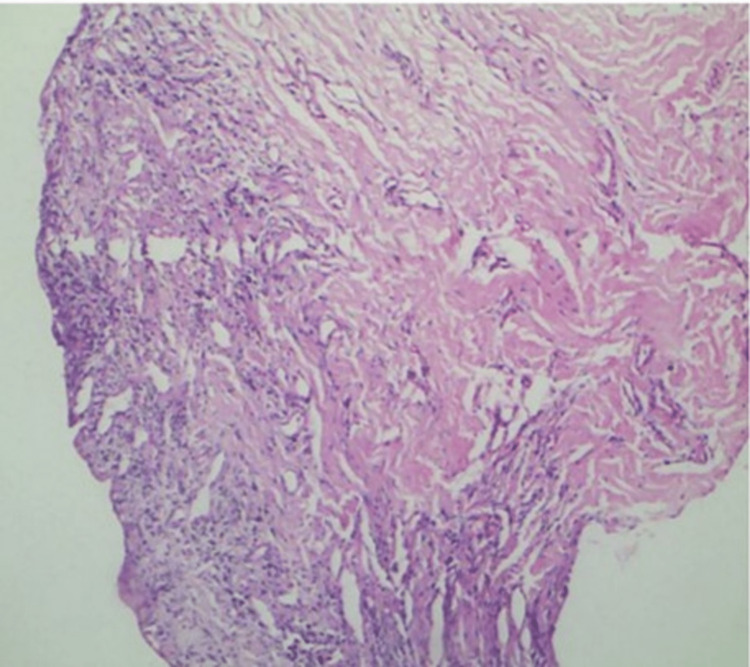
Hematoxylin and Eosin staining on 10x original magnification

A 65-year-old female presented with redness and swelling of gums that started around two months after the administration of amlodipine and was progressing since then. The patient was a recently diagnosed case of hypertension and was started on oral administration of amlodipine (5mg), a calcium channel blocker, for the past two months. The gingival lesions were associated with pain that was stabbing in nature causing difficulty in chewing and resulting in mal-alignment of the teeth. Dermatological examinations revealed erythema and induration of the gingival tissue, with generalized enlargement of the tissue extending up to marginal and interdental gingiva (Figure 1). The probing depth of the gingival sulcus was between 3mm to 5mm. General and systemic examinations were within normal limits. Routine blood culture and urine investigations were within normal limits. Histopathological examinations of the lesions revealed, mucosal erosion along with the dermis depicting hypervascularity with lymphocytosis and neutrophilia. Deep dermis revealed thick and haphazardly arranged collagen fibres (Figure 2). On the basis of the findings, the diagnosis of inflammatory gingival hypertrophy was made. Immediately, the patient was referred to a physician and the administration of amlodipine was ceased. She was shifted to the alternate class of anti-hypertensives. Within a few days, the progress of the disease was halted.

## Discussion

A second-generation dihydropyridine calcium channel blocker called amlodipine, when administered with a dose of 5mg, has been linked to gingival hypertrophy. According to studies, the incidence of amlodipine-induced gingival hypertrophy ranges from 1.7% to 3.3%. The prevalence associated with calcium channel blocker therapy may be as high as 38% [8,9]. Men are 3.3 times more likely to develop gingival hypertrophy when compared to women [3,10]. Anticonvulsants, immunosuppressants (like cyclosporine), and calcium channel blockers (like nifedipine) have all been linked to systemic drug-induced gingival hypertrophy as a potential adverse effect [11]. Additionally, there are case reports on gingival overgrowth linked to trimethoprim sulphathiazole and erythromycin. The anticonvulsants phenytoin, vigabatrin, sodium valproate, primidone, and phenobarbital are most frequently associated with gingival hypertrophy. The calcium channel blockers nifedipine, amlodipine, verapamil, nicardipine, nitrendipine, oxodipine, felodipine, and diltiazem are most often linked to gingival hypertrophy [12]. Although the mechanisms of action may vary, every medicine can cause drug-induced gingival expansion that is visible clinically and under a microscope. Within three months of initiation of the above-mentioned medications, gingival hypertrophy manifests as a hard, nodular expansion of the interdental papilla that is restricted to the gingiva's keratinized areas [2]. Since all lesions are distinguished by an increase in the connective tissue component, gingival fibroblasts are the target cell [13]. Additionally, gingival inflammation appears to be a significant risk factor for this unfavourable outcome.

Our patient did not take any of the medications mentioned above concurrently throughout the time period specified. A major risk factor for the manifestation of drug-induced gingival overgrowth is poor dental hygiene [14]. It is still unclear how gingival expansion works from the inside out. But there have already been two prominent inflammatory and non-inflammatory mechanisms proposed. The non-inflammatory process that has been hypothesised comprises reduced folic acid absorption, blocked aldosterone production in the adrenal cortex, and subsequent feedback increases in adrenocorticotropic hormone level and keratinocyte growth factor [15]. It has been postulated that patients who are treated with phenytoin but do develop gingival overgrowth have fibroblasts that are abnormally susceptible to the treatment [10,12]. It has been demonstrated that these individuals' swollen gingiva includes fibroblasts with high amounts of protein production, the majority of which is collagen. It has also been suggested that the occurrence of various proportions of fibroblast subsets in each individual that demonstrate a fibrogenic response to this medicine may be responsible for determining sensitivity or resistance to pharmacologically-induced gingival expansion. Treatment often focuses on medication substitution and efficient management of regional inflammatory causes including calculus and plaque. Surgery is suggested as a last resort if these procedures don't work to reduce the growth. Despite being effective, these treatment options do not always guarantee that the lesions won't return. Any surgical intervention must be carefully considered in terms of time and necessity [4]. Surgery is typically done to address cosmetic or aesthetic issues before any functional effects are felt. The majority of instances with amlodipine gingival overgrowth needed surgery. A method that offers quick postoperative haemostasis has shown some promise for decreasing gingival enlargement: the use of carbon dioxide lasers. A laser has a stronger bactericidal and hemostatic impact than a scalpel, and it also creates a reasonably dry field that improves sight.

## Conclusions

Hypertension is one of the most notorious non-communicable disease known to the medical fraternity. Amlodipine, a calcium channel blocker, is frequently used for the treatment of the same. Drug reaction seen after administration of this drug is quite rare and presentation is scarce. Gingival hyperplasia is a distinct presentation seen. We ask the physician to be vigilant because even with a brief and low dosage administration of amlodipine, gingival overgrowth might be an adverse effect. Treatment typically focuses on drug replacement when possible and also effective management of local inflammatory variables including calcification and plaque. Surgical intervention is advised if these approaches don't work to reduce the enlargement. Although effective, these treatment options do not always stop the lesions from returning.
